# Affective coding: the emotional dimension of agency

**DOI:** 10.3389/fnhum.2014.00608

**Published:** 2014-08-12

**Authors:** Antje Gentsch, Matthis Synofzik

**Affiliations:** ^1^Research Department of Clinical, Educational and Health Psychology, University College LondonLondon, UK; ^2^Department of Neurodegenerative Diseases, Hertie-Institute for Clinical Brain Research, University of TübingenTübingen, Germany; ^3^German Research Center for Neurodegenerative Diseases (DZNE), University of TübingenTübingen, Germany

**Keywords:** agency, emotion, prediction, self-awareness, schizophrenia, cue integration, reward

## Abstract

The sense of agency (SoA) (i.e., the registration that I am the initiator and controller of my actions and relevant events) is associated with several affective dimensions. This makes it surprising that the emotion factor has been largely neglected in the field of agency research. Current empirical investigations of the SoA mainly focus on sensorimotor signals (i.e., efference copy) and cognitive cues (i.e., intentions, beliefs) and on how they are integrated. Here we argue that this picture is not sufficient to explain agency experience, since agency and emotions constantly interact in our daily life by several ways. Reviewing first recent empirical evidence, we show that self-action perception is in fact modulated by the affective valence of outcomes already at the sensorimotor level. We hypothesize that the “affective coding” between agency and action outcomes plays an essential role in agency processing, i.e., the prospective, immediate or retrospective shaping of agency representations by affective components. This affective coding of agency be differentially altered in various neuropsychiatric diseases (e.g., schizophrenia vs. depression), thus helping to explain the dysfunctions and content of agency experiences in these diseases.

## Introduction

The close relations between emotions and actions are ubiquitous during our active engagement with the world. Emotions are the force initiating and guiding behavior by making people act in certain ways in order to achieve or avoid significant outcomes, and actions in turn change how we are feeling and give rise to particular emotional states. If a person feels in control over her own body or the environment she may experience affective states of pride or guilt, and vice versa, a context of helplessness and depression may alter her predictions and perception of actions and outcomes. It is therefore surprising that the affective dimensions and components of actions have not been taken into the equations of current models of the sense of agency (SoA), i.e., of the registration that I am the initiator of my actions and related events (Gallagher, 2000; Synofzik et al., 2008a,b). The affective dimensions provide the basis for the evaluation of self-controlled actions attributed to one’s own agency, leading–for example–to feelings of personal capacity, self-esteem or relevant self-conscious emotions such as guilt, shame, pride, and embarrassment. Moreover, the affective components of our actions (e.g., affective dispositional state of the individual, affective social context, affective value of the action outcome) modulate our inclination to accept our action consequences and outcomes as caused by ourselves or not.

Here our goal is to explore from an affective perspective, what shapes our SoA. Current empirical and theoretical advances in understanding agentive self-awareness from an affective point of view will be discussed in order to stimulate future research and to suggest a necessary extension of current conceptual frameworks of agency to include the affective dimension of action. First, we briefly review recent views suggesting a tight link between emotion, action representation and self-awareness. Second, we provide a review of existing studies explicitly addressing affective influences on the SoA. Third, we discuss different affective determinants and distinguish possible mechanisms underlying the emotion-agency link, introducing the novel concept of “affective coding” of agency which might occur prospectively, immediately or retrospectively (*post-hoc*). The implications of this affective perspective for our understanding of relevant agency disorders will be discussed. We hypothesize that in particular the “affective coding” between agency and action outcomes might play a crucial role in agency processing both in health and disease.

## The role of emotion in action representation and self-awareness

Recent evidence in cognitive neurosciences suggests that action representation is strongly influenced by emotions and that several brain structures are operating in networks to integrate affectively significant signals with action cognition and relevant behavioral control processes (Pessoa and Adolphs, 2010). The general idea of a direct link between perceptual states and action representation is most familiar from common coding theory in cognitive psychology (Hommel et al., 2001) claiming that actions are represented according to their perceptual consequences. This theoretical approach has been further extended to include affective codes as being part of these action representations and essentially shaping them (Krebs et al., 2010; Eder et al., 2012). It has been shown, for example, that learning of action-effect associations can be modulated by the motivational value of an action during the acquisition phase and the motivational disposition of an individual (Muhle-Karbe and Krebs, 2012). It is worth noting, however, that goal representations associated with motivational states compared to the hedonic experience of the outcome itself might involve dissociable mechanisms and influences on action representations.

Self-awareness in general has frequently been linked to the processing of emotions and bodily states. Affective accounts of selfhood assume that basic pre-reflective forms of self-awareness are grounded in representations of emotions and bodily sensations (Damasio, 1999). This view has recently been formalized within a computational framework of “predictive processing” that links action, sensory perception and interoception (Seth et al., 2011). According to this model of “interoceptive inference”, emotion and embodied self-awareness arise from generative models predicting interoceptive signals that result as a consequence of internal autonomic control signals or environmental changes. Agency is considered to be one important predictor of changes in internal bodily states that generate interoceptive signals, for example an increase in heart rate when performing or preparing for a personally challenging action. These prediction signals are thought to give rise to a basic sense of presence and agentive awareness (Seth et al., 2012). That means that, action perception and attribution is thought to be determined not only by exteroceptive and proprioceptive cues but also by their close interplay with interoceptive bodily signals. This multi-cue integration is at the core of an increasingly influential account of agentive self-awareness, the multifactorial weighting account (Synofzik et al., 2008a, 2013; Vosgerau and Synofzik, 2012). Multiple probabilistic cues are thought to be weighted as a function of their predictive accuracy for prospective agency and integrated with action-related signals based on their reliability and salience during action execution and during retrospective processing of the action. Important explanatory gaps still remain, though, with respect to the exact mechanisms of how precisely emotional states may interact with probabilistic and action-related signals to inform feelings and judgments of agency at different levels.

Besides cognitive approaches to self-awareness, a strong motivational and emotional dimension of self-processing has been posited in psychology (Leary, 2007). A number of “self-motives” such as motives for self-enhancement, self-verification, self-expansion, or self-assessment are thought to affect action and cognition, and have been argued to function to protect people’s social well-being. These “self-motives” are thought to be strongly linked to different “self-conscious emotions”–including guilt, shame, embarrassment, social anxiety and pride–that emerge from self-representation (Leary, 2007). Experimental studies have shown that although people may prefer objective, accurate information about themselves under certain circumstances, the desire for self-enhancement or verification of pre-existing self-conceptions may override this motive (Sedikides and Strube, 1995). In line with this view, it is well known that our mind has developed ways to maintain the integrity of a positive self-concept even in contexts of failure (Mezulis et al., 2004). Ample evidence indicates the tendency in healthy individuals to make self-serving attributions by relating positive outcomes to the self and negative outcomes to others. This affective shaping of outcome attributions can be altered in different neuropsychiatric diseases; for example, it seems to be lacking in depression (Alloy and Abramson, 1979). These findings can already be taken as first evidence for that fact that the selection of new self-relevant information might follow a differential weighting whereby some cues are weighted more strongly than others (e.g., positive or “self-serving” cues are weighted more strongly than negative or self-detrimental cues) (Synofzik et al., 2009b). Yet this weighting might not always follow the rules of an statistical optimal cue integration, namely the reduction of uncertainty about the self as a cause of sensory input by giving most weight to the objectively most reliable cues, as would be suggested by optimal cue integration accounts (Synofzik et al., 2009b, 2013).

## Affective influences on the sense of agency

Based on the above mentioned lines of evidence it is reasonable to generally assume a tight link between emotions and processes underlying agency registration. However, current accounts of the SoA are primarily computational cognitive models, grounded in constructs of motor control theory, without the need for emotional states to be taken into account (Wolpert et al., 1995). Accordingly, the SoA is thought to depend on predictive cues derived from internal forward modeling of upcoming sensory action consequences in the motor system (Frith et al., 2000b). Following first critique of these models as accounts of agency (Synofzik et al., 2008a), a growing body of literature has now started to extend this view by highlighting the importance of a *combination* of *different* cues weighted according to their reliability to signal agency (Moore et al., 2009; Synofzik et al., 2010; Desantis et al., 2012b). Recent models assume a multifactorial weighting process based on some form of Bayesian optimal cue integration (Fletcher and Frith, 2009; Synofzik et al., 2009b; Moore and Fletcher, 2012). However, these models still largely spare out the contribution of emotional and motivational mechanisms, and only recently has empirical work begun to explicitly address the affective influences on specific sensorimotor markers of agency (see also, Synofzik et al., 2013).

Several emerging levels of evidence point toward the importance of emotional influences on both functional and dysfunctional agentive processing. A well-studied phenomenon reflecting the affective influence on agency experience is the “self-serving bias”. This refers to the pervasive tendency of healthy individuals to make self-favoring causal attributions when facing significant positive or negative outcomes (Greenberg et al., 1982; Mezulis et al., 2004). Specifically, people tend to attribute causes of positive outcomes more often to internal factors and negative outcomes more often to external factors. This seems to reflect a mechanism for maintaining self-esteem and reducing cognitive dissonance (Harmon-Jones et al., 2009). Clinically depressed patients typically exhibit the inverse pattern of this bias, a “depressive attributional style”, reflected in the internalization of responsibility for negative events and externalization of agency for positive events (Alloy and Abramson, 1979).

This evidence for the existence of self-serving attribution biases is based on explicit, retrospective self-report, thus indicating that affective modulation occurs on the level of *judgment* of agency (Synofzik et al., 2008a). These reports are now complemented by recent findings demonstrating that the affective value of action outcomes already influences also the low-level sensorimotor representations of actions and agency in a self-serving way, i.e., the *feeling* of agency (Synofzik et al., 2008a). For example, it was found that participants’ perception of pointing actions is biased towards positive and away from negative outcomes (Wilke et al., 2012). Other studies observed reduced temporal binding between actions and consequences signaling monetary loss (Takahata et al., 2012) or eliciting negative emotional vocalizations (Yoshie and Haggard, 2013). These findings suggest the existence of automatic valence specific effects of emotions on implicit low-level measures of the SoA. However, they also have to be interpreted with caution as—in contrast to a long-standing assumption—intentional binding does not necessarily reflect a signature of agency. As we have argued earlier (Synofzik et al., 2009a), the fact that perceived time intervals between movement and effect were decreased by priming also in case of involuntary movements opens up the possibility that the binding between movement and effect might not be specific to agency and intentionality, but can also present—at least in part—a more unspecific effect linked to temporal binding between two external events (in this case between the two congruent sounds, i.e., between prime and effect). Indeed, recent studies suggest that intentional binding is neither linked specifically to motor predictive processes (Desantis et al., 2012a; Hughes et al., 2013) nor to agency (Buehner and Humphreys, 2009; Buehner, 2012; Dogge et al., 2012), but rather to causality in general. However, even if the phenomenon of binding of movements to their effects was not specifically linked to agency, it could still contribute to the experience of agency, for instance, by accentuating subject’s perception of the temporal contiguity between movements and their effects (Desantis et al., 2012a).

Notably, any observed emotional modulation of these low-level measures of action perception and SoA could in principle be mediated by predictive influences as well as postdictive reconstruction of the experience (Synofzik et al., 2013). Future studies are needed to clearly modulate only one of these two factors. Alternatively, they could examine valence effects specifically at the early stages of anticipation and outcome processing in order to disentangle predictive and reconstructive components (e.g., by using the high temporal resolution of EEG). Predictive cues are assumed to be weighted according to their reliability to indicate the most likely outcome (Moore et al., 2009; Synofzik et al., 2010). However, cue weighting may further be influenced by activated self-motives in a given social/emotional context. This view is supported by the empirical picture of self-serving biases, which is rather consistent with respect to the tendency to attribute success to the self (“positive bias”), but mixed with respect to the tendency to attribute failure (“negative bias”). It has been argued that this is due to the “negative bias” being moderated by additional self-motives such as self-assessment and self-improvement and the perceived capacity to do so (Duval and Silvia, 2002). Moreover, the weighting of affective predictions and the perception of emotional valence of action outcomes could be affected by the emotional and attentive state of the individual, and may be critically altered in certain psychopathological conditions marked by distorted agency experience, which will be addressed in the following.

## Emotions in agency disorders

Psychopathology research provides abundant evidence for a strong interrelation between emotion and action, suggesting that aberrant sensorimotor awareness could be rooted in deficient emotional processing of action-related signals. In affective disorders, such as mania and depression, action awareness abnormalities are at the core of the phenomenological expression of these disorders. At explicit levels, self-awareness is often dramatically altered towards grandiose delusions and inflated sense of power in periods of mania (Knowles et al., 2011), or towards a depressive realism in depressive episodes (Alloy and Abramson, 1979). Previous studies suggest that already in healthy individuals showing dysphoric compared to non-dysphoric affective states the experience of self-agency and self-serving attributions are reduced (Aarts et al., 2006). Moreover, for depression the possibility has been raised that impaired action monitoring may represent an important depressive endophenotype (Olvet and Hajcak, 2008; Holmes et al., 2010), as reflected for example in impaired post-error behavioral adaptation (Holmes and Pizzagalli, 2008). The role of these monitoring abnormalities for the attenuated self-serving biases in action awareness in these patients, however, remains unclear.

Another indication for emotional influences on agentive awareness comes from neurological patients with anosognosia for hemiplegia (AHP), which can show delusional experience of self-agency despite a complete lack of voluntary movement after brain lesion (Feinberg et al., 2000). These patients may claim that they can move on request or provide excuses (confabulations) for not moving, and some may even believe to have moved ignoring visual, proprioceptive and external cues signaling the absence of an action. Besides models assuming deficits in sensorimotor mechanisms (Heilman et al., 1998; Frith et al., 2000b; Berti et al., 2005), emotion-related explanations have been put forward, stressing the role of motivational factors and emotion regulation mechanisms in generating the unawareness and higher-order confabulations (Vuilleumier, 2004; Turnbull et al., 2005; Fotopoulou, 2010). It has been noted that transient episodes of improved action awareness in these patients are accompanied by an increase in depressive symptoms (Kaplan-Solms and Solms, 2000; Fotopoulou, 2010). AHP patients seem to fail to integrate negative emotions with explicit self-awareness (Fotopoulou et al., 2010). Moreover, recent evidence shows that negative (but not positive) performance feedback can cause improved action awareness in these patients (Besharati et al., submitted). Based on neuroimaging studies reporting damage in anterior parts of the insula (Berti et al., 2005; Karnath et al., 2005), it has been argued that a lack of re-representation of emotional action-related information may lead to the abnormally preserved self-agency experience in these patients (Fotopoulou et al., 2010). It still remains to be explored, however, to which extent this impairment can explain the variations in the clinical presentations of AHP including accompanying confabulations and delusional beliefs around agency and ownership.

Delusions of control in schizophrenia are often seen as the paradigmatic case of a disrupted SoA, and they have typically been explained as motor-cognitive phenomena without relation to emotional and motivational processes (Frith et al., 2000a). However, these frameworks fail to provide an explanation for the often emotionally tuned semantic content and context of delusions in schizophrenia, including delusions of control. Although studies focusing specifically on the thematic content of delusions of influence in schizophrenia patients are still missing, studies analyzing delusions in schizophrenia in general have shown that these refer often not to trivial, non-emotional actions in daily life (e.g., brushing teeth or typing on a computer), but to actions and contexts with high affective and/or moral value, including thematic contents of religion, sex, grandiosity, persecution, and guilt (Frith, 1992; Linskey, 1994; Suhail, 2003). Here the affective and moral valence gains major influence on both the sensorimotor and the cognitive level, such that the action experience and possibly also the action attribution is altered. Many experimentators so far have used mainly simplified non-affective actions (e.g., simple joystick movements (Spence et al., 1997) or simple pointing movements (Synofzik et al., 2010)) to experimentally test and operationalize action monitoring deficits, which they then tried to correlated with the patients’ psychopathology of delusions of controls. This testing and operationalization strategy should, of course, not be mistaken as an indicator that the thematic content of the patients’ psychopathology *per se* would entail such simple movements.

## Affective coding of agency: how affect may influence the sense of agency

**Figure 1:**
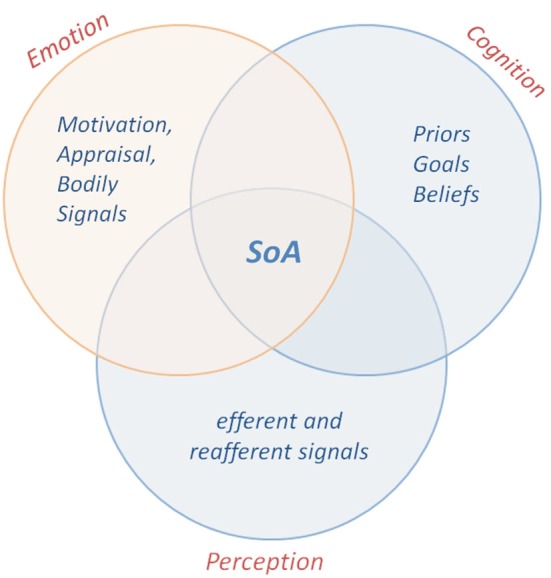
**Illustration of the integration of the affective dimension in cognitive-sensorimotor mechanisms underlying agentive awareness**. The contribution of emotional cues in synchrony with sensorimotor and cognitive cues in the formation of sense of agency (SoA) is displayed.

We suggest “*Affective Coding of Agency”* as an essential extension of current cue integration models of agency. Emotions interact with agency in manifold ways, given the different levels and aspects of emotion representations and the various possible mechanisms mediating the interplay between emotion and action awareness. We hypothesize that both the expected and actual valence of an action outcome act as strong agency cues in synchrony with cognitive and sensorimotor coding of actions (Figure 1).

### Emotional determinants of agency

**Figure 2:**
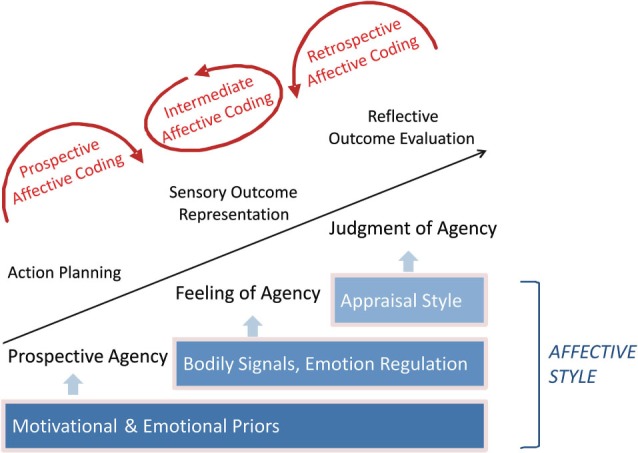
**Illustration of the influence of distinct emotional determinants at different stages of agency processing**. At the first stage of action planning, priors derived from affective state, affective trait or affective context variables influence prospective representations of agency (prospective affective coding). At the second stage, feelings of agency can be shaped by rapid appraisal of emotionally salient information and emotional bodily responses (intermediated affective coding). Thirdly, positive or negative self-schemas and self-enhancement or self-protection motives may guide *post-hoc* explicit attributions of agency (retrospective affective coding). Finally, individual differences in the degree of emotion regulation during an affective state (affective style) may moderate the interplay between emotion and agency at all three levels of representation.

Due to the multifaceted nature of emotions, different components of emotions determine agency processing at different stages. Specifically, emotions can influence agency at the stage of (i) prospective agency; (ii) the immediate feeling of agency; and (iii) the *post-hoc* judgement of agency (Figure 2).

1. ***Prospective affective coding***. Emotional and motivational priors of a subject’s individual state of an action may strongly shape prospective representations of agency (for example, his depressed vs. euphoric mood or his open-minded vs. buttoned-up attitude; or his positive vs. negative expectation on the affective outcome of an action; or his high vs. low motivation to perform the upcoming action). Also the affective dimensions of the specific background and context of an action will prospectively shape the agency experience (for example, acting in a friendly vs. hostile environment). This prospective process can be called “prospective affective coding” of agency (Figure 2).
2. ***Immediate affective coding***. Fast and automatic emotion processes (LeDoux, 1996) are reflected in immediate emotional bodily responses and early mechanisms of sensory gating based on internal bodily and motivational states (Vuilleumier, 2005; Pourtois et al., 2013). They may construe the immediate pre-reflective feeling of the action (Seth et al., 2011; Synofzik et al., 2013) which is neither fully determined by affective priors nor by the affective *post-hoc* evaluation of the action. Interindividual differences in interoceptive sensitivity may be an important mediator at this level. This immediate shaping of agency by direct affective processes can be called “immediate affective coding” of agency (Figure 2).
3. ***Retrospective affective coding***. At the stage of the *post-hoc* evaluation of an action (which might also often occur in rather immediate and automatic manner in everyday life), agency is shaped by the affective appraisal of the actual action outcome (Wilke et al., 2012). Also individual attributional styles as implied by the depressive realism hypothesis (Alloy and Abramson, 1979) and situational self-schema and self-motive activation may influence these *post-hoc* judgments about self-agency (Aarts et al., 2006). This affective *post-hoc* shaping of agency can be called “retrospective affective coding” of agency.

Some affective factors might present general determinants of agency and run across all three different stages of affective agency shaping, modulating all three of them. One of these general determinants might be individual differences in “affective style”, that is, the tendency for regulating emotions. Strategies of behavioral re-adjustments, affect suppression or tolerance could also be important general mediators of affective coding of agency. For example, a core feature of depersonalization disorder, self-detachment including a lowered SoA, has been proposed to result from a “shutting down” of emotional responses due increased fronto-insula/limbic inhibitory regulation (Sierra and Berrios, 1998; Phillips et al., 2001).

## Conclusion

Bringing the affective quality of actions into the empirical picture will provide an important extension to current theoretical accounts of agency experience, and will do justice to the individual differences and pathologies in feelings of self-control. Why do some people have immediate feelings of self-efficacy and others do not when facing the same outcomes? And how deep-rooted are these feelings in embodied social knowledge and actual behavior towards the environment? Self-motives may find their way into an embodied signature by shaping the weight of our predictive codes and the gates through which we perceive the external world. For example, most recent conceptualizations of predictive models hold that the influence of prediction on perception critically depends on the assignment of salience based on dopaminergic neuromodulation of attentional processes (Friston et al., 2012). The degree of self-serving affective biases in agentive awareness may respectively depend on increased attentional resources directed to expected favorable outcomes compared to unfavorable outcomes. For example, one way to regulate emotion or to maximize positivity of the self-concept is through selective withdrawal of attention to unexpected unfavorable outcomes during self-action leading to attenuated outcome salience and reduced belief updating for unfavorable self-generated events. However, the precise nature of salience-weighted perceptual inference in relation to emotions will have to be specified in considerable more detail to understand its contribution to agentive self-awareness. A systematic investigation of discrete aspects of affective processes and emotional regulation strategies could prove a promising avenue in this direction. Importantly, the relation between emotion and agency is bi-directional rather than uni-directional and the concurrent investigation of reciprocal relations between emotion and action awareness at the neural and cognitive level will be the challenge for future investigations.

## Conflict of interest statement

The Reviewer Andrea Desantis declares that, despite being affiliated to the same institution as author Antje Gentsch, the review process was handled objectively and no conflict of interest exists. The authors declare that the research was conducted in the absence of any commercial or financial relationships that could be construed as a potential conflict of interest.
